# Clinical significance of repeat rebiopsy in detecting the EGFR T790M secondary mutation in patients with non-small cell lung cancer

**DOI:** 10.18632/oncotarget.25705

**Published:** 2018-06-29

**Authors:** Eiki Ichihara, Katsuyuki Hotta, Toshio Kubo, Tsukasa Higashionna, Kiichiro Ninomiya, Kadoaki Ohashi, Masahiro Tabata, Yoshinobu Maeda, Katsuyuki Kiura

**Affiliations:** ^1^ Department of Allergy and Respiratory Medicine, Okayama University Hospital, Okayama, Japan; ^2^ Center for Innovative Clinical Oncology, Okayama University Hospital, Okayama, Japan; ^3^ Center for Clinical Oncology, Okayama University Hospital, Okayama, Japan; ^4^ Department of Clinical Pharmaceutics, Okayama University Hospital, Okayama, Japan; ^5^ Department of Hematology, Oncology and Respiratory Medicine, Okayama University Graduate School of Medicine, Dentistry and Pharmaceutical Sciences, Okayama, Japan

**Keywords:** EGFR, lung cancer, osimertinib, rebiopsy, T790M

## Abstract

**Background:**

Osimertinib is an essential drug to treat non-small-cell lung cancer (NSCLC) harboring the epidermal growth factor receptor (EGFR) T790M mutation, and rebiopsy is necessary to detect this mutation. However, the significance of repeat rebiopsy in NSCLC patients whose first rebiopsy was T790M-negative remains unclear. We used a retrospective cohort to clarify this issue.

**Methods:**

We reviewed the medical records of patients with NSCLC harboring EGFR mutations who underwent EGFR tyrosine kinase inhibitor (TKI) treatment at Okayama University Hospital between January 2015 and January 2017.

**Results:**

Of 102 patients with EGFR-mutant NSCLC, 55 underwent rebiopsy after acquired resistance to prior EGFR TKIs. Pre-existing activating EGFR mutations were found in all 55 rebiopsied samples. Of the 55 samples, 25 were T790M-positive (45%). Among the remaining 30 patients (T790M-negative on the first rebiopsy), 21 underwent additional rebiopsies following interval therapy. Of the 21 patients, 11 were T790M-positive on the second rebiopsy and 1 on the third. We also evaluated the efficacy of osimertinib in patients who needed a repeat rebiopsy to detect the T790M mutation. Osimertinib showed good activity with an objective response rate of 50%.

**Conclusions:**

Repeat rebiopsy increases the ability to detect a secondary mutation (T790M) in EGFR.

## INTRODUCTION

Lung cancer is a leading cause of cancer deaths in Japan and worldwide. The discovery of mutations in the epidermal growth factor receptor gene (*EGFR*) in non-small-cell lung cancer (NSCLC) patients has dramatically changed the treatment strategy [1]. The efficacy of EGFR tyrosine kinase inhibitors (TKIs) to treat EGFR-mutant NSCLCs is dramatic, with a 70% objective response rate (ORR), 1 year progression-free survival (PFS), and more than 2 years overall survival (OS) [2, 3]. The Achilles heel of EGFR TKIs is acquired resistance, which inevitably occurs within 1–2 years of treatment. The EGFR secondary T790M mutation accounts for half of the resistance mechanisms [4, 5]. Osimertinib, a third-generation EGFR TKI, overcomes this resistance. This potent EGFR TKI shows a 61% response rate in patients with T790M-positive NSCLC, who acquired resistance to a prior EGFR TKI, but only 21% in those without the T790M mutation [6]. An international phase 3 trial compared osimertinib with platinum-pemetrexed doublet for treating patients with T790M-positive NSCLC who acquired resistance to a prior EGFR TKI [7]. In that study, the median PFS was significantly longer in patients treated with osimertinib than in those treated with platinum therapy plus pemetrexed (10.1 vs. 4.4 months; hazard ratio [HR], 0.30; 95% confidence interval [CI], 0.23–0.41). Osimertinib is now an essential drug to treat patients with T790M-positive NSCLC who acquired resistance to a first/second-generation EGFR TKI.

T790M-positive patients can expect prolonged inhibition of tumors during treatment with osimertinib, whereas treatment options are limited for T790M-negative patients. Most T790M-negative patients receive chemotherapy or best supportive care, depending on their condition. However, the ORR of chemotherapy is only 20% in contrast to that of osimertinib [7]. These findings imply that the presence of T790M has a great impact on the subsequent clinical course of patients, leading to better outcomes. However, the significance of repeat biopsies in patients with NSCLC who are T790M-negative on an initial rebiopsy is unclear. In addition, assuming that rebiopsy finds additional T790M-positive patients, the efficacy of osimertinib in such patients remains unclear.

These unresolved questions prompted us to investigate whether multiple rebiopsies following some interval could detect additional T790M-positive NSCLC patients and whether osimertinib would be effective in such patients.

## RESULTS

### Patient characteristics

Among 102 patients with EGFR-mutant NSCLC treated with EGFR TKIs, 55 who underwent at least one rebiopsy to detect the T790M mutation were included in the analysis. The patients’ characteristics are summarized in Table 1. Their median age was 66 years (range, 41–85 years), and female patients were dominant. All patients had adenocarcinoma histology. In total, 35 patients had the *EGFR* exon 19 deletion and 20 had the exon 21 L858R point mutation.

**Table 1:** Patient characteristics

Median age, years (range)	66 (41-85)
Histology (Ad / others)	55/ 0
*EGFR* mutation status (del / L858R)	35/ 20
Smoking, median pack-years (range)	0 (0-108)
Gender (male / female)	17 / 38
Stage (IIB or IV / recurrence)	38 / 17

Abbreviations: Ad, adenocarcinoma.

del, exon 19 deletion mutation; L858R, exon 21 L858R point mutation.

### Frequency of the secondary T790M mutation

**Figure 1:**
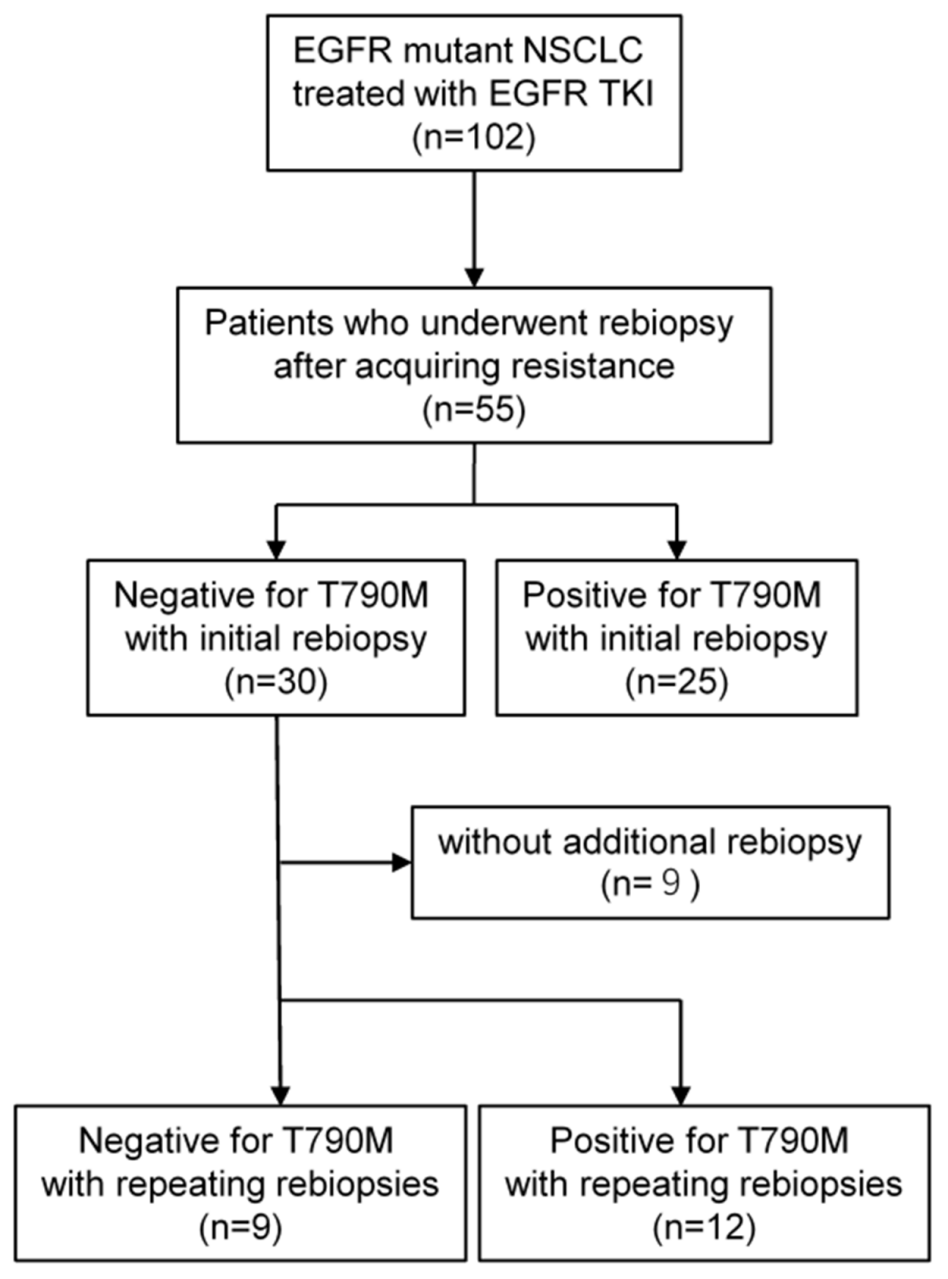
Study flow chart

Figure 1 presents a flow chart of the patients included in this study.

Among the total of 102 patients, 47 had no successfully obtained rebiopsy sample (31 without progression, 9 with progression before clinical approval of osimertinib, 6 unsuccessful tissue rebiopsies or no targetable lesion, and 1 excluded for sequential osimertinib due to drug-induced pneumonitis caused by prior EGFR TKI).

Among the 55 patients who underwent the first rebiopsy, 25 (45.4%) were diagnosed as T790M-positive. Of the remaining 30 patients, 21 underwent repeat rebiopsy following interval treatment, and 12 additional patients were diagnosed as T790M-positive (12/55, 21.8%). In total, the frequency of T790M increased from 45.4% (25/55) to 67.3% (37/55) by repeat biopsy.

**Figure 2:**
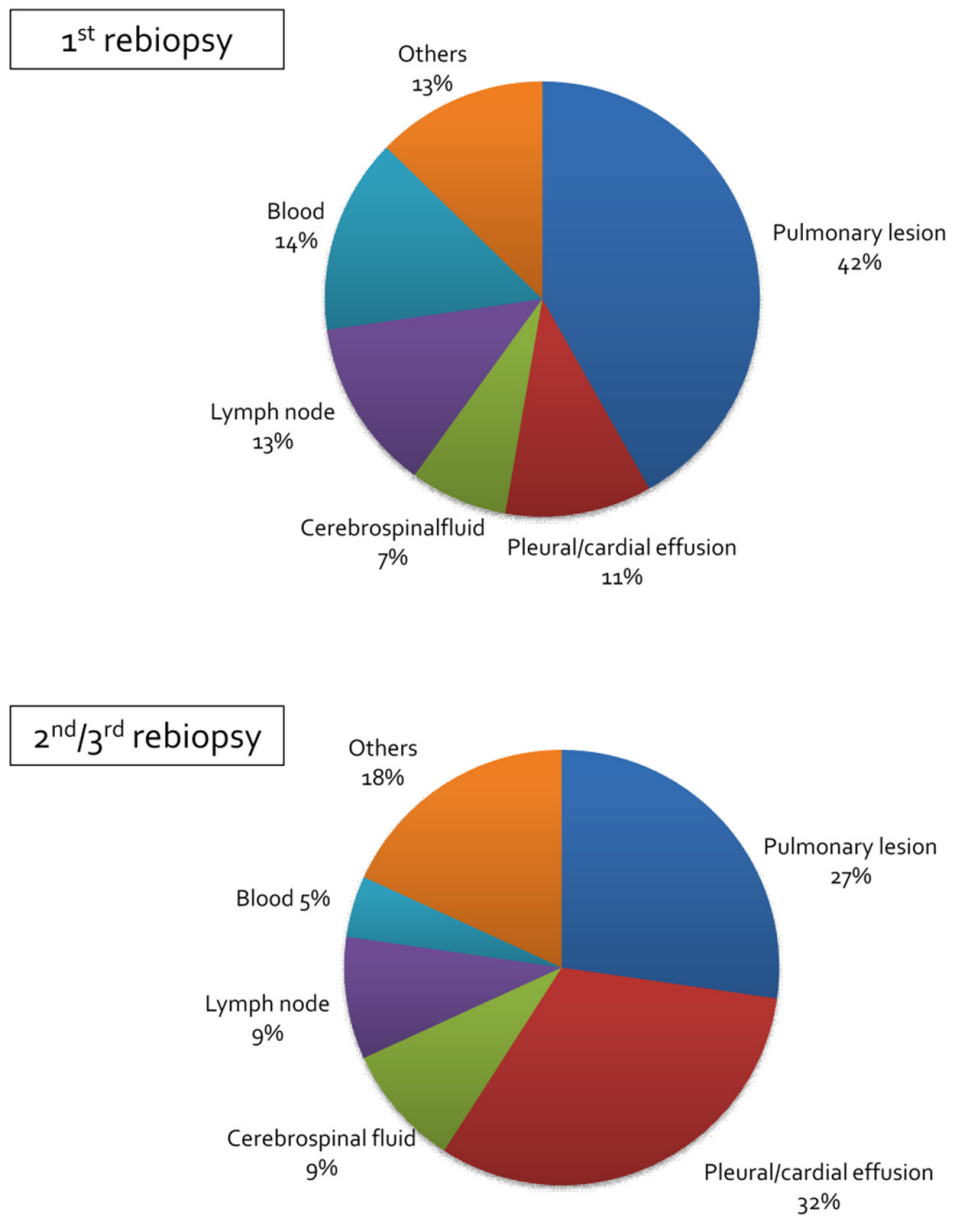
Rebiopsied lesion Each pie chart shows the lesion where a rebiopsy was performed.

The rebiopsied sites are summarized in Figure 2. In total, 42% of first rebiopsies were performed on pulmonary lesions, followed by blood biopsy, and lymph-node biopsy, while the frequency of pleural or pericardial effusion increased in the second/third rebiopsies. Because fewer incidents of T790M in central nervous system lesions are reported [8, 9], we determined the T790M incidence using cerebrospinal fluid; only one T790M patient was found using cerebrospinal fluid. T790M incidence, detected using cerebrospinal fluid and other samples, was 0/3 and 25/52 in the first rebiopsy, and 1/2 and 11/21 in the 2^nd^/3^rd^ rebiopsy, respectively. We also explored whether any patients were T790M negative using a blood-based assay during the first rebiopsy and T790M positive using a tissue-based assay during the second or third rebiopsy. No patient was blood-negative and tissue-positive, implying that the T790M-positive repeated rebiopsy results in this cohort were not caused by lower sensitivity of the blood-based assay.

### Response to osimertinib

**Figure 3:**
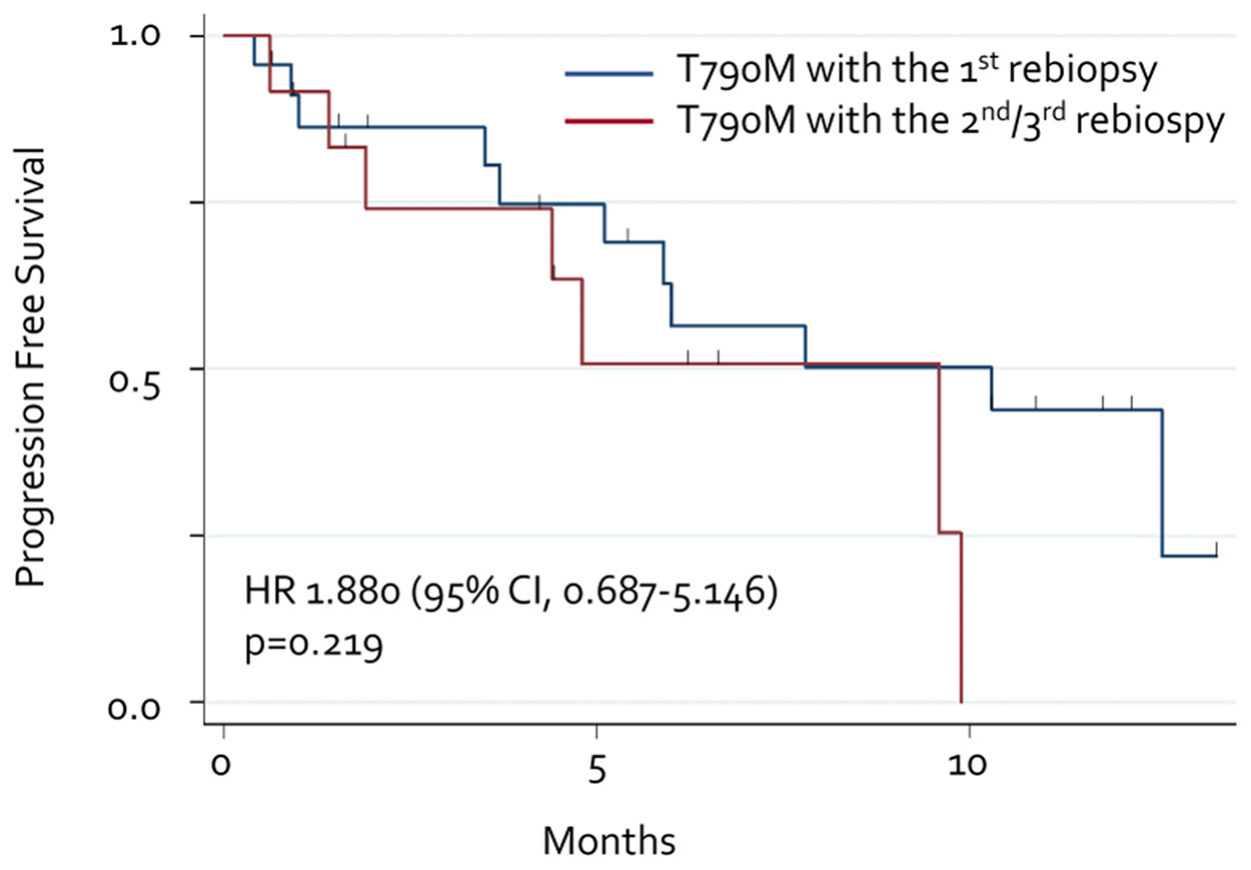
Progression-free survival with osimertinib Each line represents the progression-free survival curve for osimertinib. Blue line: T790M detected by initial rebiopsy. Red line: T790M detected by repeated rebiopsy.

We compared the efficacy of osimertinib in patients with the T790M mutation diagnosed by second/third rebiopsy with that in patients diagnosed by the first rebiopsy. The response to osimertinib was evaluated in 18 patients with the T790M mutation detected by the first rebiopsy and 10 with the T790M mutation detected by repeat rebiopsy. The ORRs were 56% and 50%, respectively (Table 2.) We also compared the Kaplan-Meier curves in both groups, but no significant difference was observed between them (HR, 1.880; 95% CI, 0.687–5.146; p = 0.219; Figure 3).

**Table 2:** Response to osimertinib

	T790M detected by	
	1^st^ rebiopsy N=18	2^nd^/3^rd^ rebiopsy N=10
CR	0 (0%)	0 (0%)
PR	10 (56%)	5 (5%)
SD	6 (33%)	2 (20%)
PD	2 (11%)	3 (30%)
**ORR**	**56%**	**50%**

### Characteristics of the patients with the T790M mutation detected by repeat rebiopsy

Finally, we determined the characteristics of the 12 patients in whom the T790M mutation was detected by a second/third rebiopsy (Table 3). The median interval between the rebiopsies was 8.3 months. Seven of the twelve patients had rebiopsies of different lesions from the first rebiopsy. Five patients underwent EGFR TKI therapy between the rebiopsies, while the remaining seven did not.

**Table 3:** Characteristics of the patients with the T790M mutation detected by repeat biopsy

*EGFR* mt del /L858R	9 / 3
Rebiopsies from the same lesion yes/no	5 / 7
Interval between rebiopsies, month	8.3 (2.2 – 21.8)
TKI treatment between rebiopsies yes /no	5 / 7

## DISCUSSION

We investigated the significance of repeat biopsy in detecting the T790M mutation in NSCLC patients with acquired resistance to EGFR TKIs. In our cohort, T790M mutation would have been missed in 12 (21.8%) of 55 patients without multiple rebiopsies, and subsequent rebiopsies increased the frequency of finding T790M mutation from 45.8% to 67.3%. In other words, 12 of 55 patients (21.8%) would have missed the chance to be treated with osimertinib without the additional rebiopsy, implying the importance of repeating a rebiopsy even in patients who did not have the T790M mutation according to the first rebiopsy.

It remains unclear why the T790M mutation was found in a repeat biopsy in patients who were T790M-negative on the first rebiopsy. T790M might have been acquired *de novo* between the rebiopsies [10]. Actually, five of the twelve patients who needed an additional rebiopsy to detect the T790M mutation received EGFR TKI therapy between rebiopsies. A sub-population harboring the T790M mutation might have been selected for interval EGFR TKI therapy. However, the remaining seven patients did not receive any EGFR TKI in the rebiopsy interval. Given that patients treated with medications other than EGFR TKIs are unlikely to select or develop the T790M mutation, *de novo*-acquired T790M does not explain those patients. Another explanation is tumor heterogeneity [11]. Many data imply intra- and inter-tumor heterogeneity, and that tumors are genetically heterogeneous even within a patient. Five of twelve patients received a repeat rebiopsy at a different site from the first one (Table 3). There may have been inter-tumor T790M heterogeneity between the first rebiopsied site and the repeat site in those patients. Intra-tumor heterogeneity could have existed in the seven patients who underwent rebiopsies from the same lesion (Table 3).

It has recently been reported that osimertinib has high efficacy for treating naïve NSCLC harboring an EGFR-activating mutation. In that study, osimertinib resulted in significantly longer PFS compared to the first-generation EGFR TKIs gefitinib and erlotinib (18.9 vs. 10.2 months). Based on the present results, osimertinib will be a first-line treatment option for advanced EGFR-mutant NSCLC. However, it is still unclear whether first-line osimertinib would show better OS, and there are significant arguments regarding which is the better strategy, osimertinib first or another EGFR TKI followed by osimertinib. Furthermore, there have been no direct comparisons between osimertinib and second-generation EGFR TKIs such as afatinib and dacomitinib. Thus, it remains important to detect an EGFR secondary T790M mutation to use the strategy with a first/second-generation EGFR TKI followed by osimertinib.

This study has some limitations. This study retrospectively analyzed heterogeneous data with a small sample size, meaning that the results are more speculative and not definitive. This issue could have biased the present findings, so our results should be interpreted cautiously.

In conclusion, this study demonstrates that some patients require multiple rebiopses. Repeat biopsy should be considered in NSCLC patients who are T790M-negative on initial rebiopsy, given that it is important to demonstrate the existence of T790M in NSCLC patients who acquire resistance to a first/second-generation EGFR TKI.

## MATERIALS AND METHODS

### Patients and study design

To detect the T790M mutation, we retrospectively reviewed the medical records of all patients with NSCLC and *EGFR* mutations who received a rebiopsy at the Department of Respiratory Medicine at Okayama University Hospital, Okayama, Japan, between January 2015 and January 2017. This study was approved by the Institutional Review Board of Okayama University Hospital (no. 1703-049).

### Rebiopsy

Rebiopsies without histological and cytological confirmation of malignancy were not excluded from the analysis. All types of rebiopsy were included in the analysis, including bronchoscopic biopsy, surgical biopsy, cerebrospinal /pericardial/pleural puncture, and liquid biopsy from blood samples.

### Detection of EGFR T790M mutation

Using tissue samples, EGFR T790M mutation status was assessed using the polymerase chain reaction (PCR) clamp method or the Scorpion amplified refractory mutation system method, both of which have been approved in Japan. The cobas EGFR Mutation Test assay (Roche Molecular Systems, Inc.) was used to assess T790M in liquid biopsies.

### Response assessment

Responses were re-evaluated in this study by two investigators, according to the Response Evaluation Criteria in Solid Tumors, version 1.1.

### Statistical analysis

PFS was defined as the period from the beginning of treatment to the day of disease progression or death from any cause using the Kaplan–Meier method. Groups were compared using the log-rank test. Significance was determined at a level of p < 0.05.
